# Distal biceps tendon rupture: advantages and drawbacks of the anatomical reinsertion with a modified double incision approach

**DOI:** 10.1186/s12891-018-2278-1

**Published:** 2018-10-10

**Authors:** L. Tarallo, M. Lombardi, F. Zambianchi, A. Giorgini, F. Catani

**Affiliations:** Orthopaedics and Traumatology Department, University of Modena and Reggio Emilia, Via del Pozzo 71, 41124 Modena, Italy

**Keywords:** Distal biceps lesion, Tendon rupture, Biceps reinsertion, Double incision

## Abstract

**Background:**

Distal biceps tendon rupture occurs more often in middle-aged male population, involving the dominant arm. In this retrospective study, it’s been described the occurrence of the most frequent adverse events and the clinical outcomes of patients undergoing surgical repair of distal biceps tendon rupture with the modified Morrey’s double-incision approach, to determine better indications for patients with acute tendon injury.

**Methods:**

Sixty-three patients with acute distal biceps tendon rupture treated with a modified double-incision technique between 2003 and 2015 were retrospectively evaluated at a mean 24 months of follow-up. Clinical evaluation including range of motion (ROM) and isometric strength recovery compared to the healthy contralateral side assessment, together with documentation of nerve injury, was performed. Patients were asked to answer DASH, OES and MEPS scores.

**Results:**

The ROM recovery showed excellent results compared to the healthy contralateral side.

The reported major complications included: one case of proximal radio-ulnar synostosis, 3 cases of posterior interosseous nerve (PIN) palsy and one case of a-traumatic tendon re-rupture. Concerning minor complications, intermittent pain, ROM deficiency < 30° in flexion/extension and pronation/supination, isometric flexion strength deficiency < 30% and isometric supination strength deficiency < 60%, lateral antebrachial cutaneous nerve (LACBN) injury, were observed. The average DASH score was 8.5; the average OES was 41.5 and the MEPS was 96.3.

**Conclusion:**

The Morrey modified double-incision technique finds its indication in young and active patients if performed within 2 weeks from injury. If performed by experienced surgeons, the advantages can exceed the drawbacks of possible complications.

## Background

Distal biceps tendon rupture is a relatively uncommon injury, representing the 3% of all tendon lesions. It is predominantly affecting middle-aged, active men [1, 2]. Typically, the injury mechanism is represented by an eccentric muscle contraction against a heavy load in a semi-flexed position [2, 3].

At clinical examination, patients report acute pain in the cubital fossa and present edema, ecchymosis and palpable tendon defect on the volar side of the elbow. The Hook sign is usually positive. False negative is possible if the lacertus fibrosus is intact. Reduced strength in forearm supination and elbow flexion is usually observed [4]. Non-operative management of these injuries has been described, but significant strength reduction in flexion and supination often occurs in these patients. Therefore, such option is not suitable in young and demanding patients. On the other hand, surgical management of distal biceps tendon ruptures can be complicated by heterotopic ossification, tendon re-rupture, superficial wound infection, synostosis and nerve injury to the lateral antebrachial cutaneous (LABC) nerve, anterior interosseous nerve (AIN), posterior interosseous nerve (PIN), median, radial and ulnar nerves [5–10].

Several techniques have been described for distal biceps tendon repair, including single anterior incision [11], often complicated by a high incidence of radial nerve palsy [12], double incision techniques exposing the radial tuberosity and allowing a smaller anterior approach, often complicated by frequent post-operative proximal radio-ulnar synostosis [13]. Others have also described a modified double-incision technique, introducing a muscle-splitting approach through the digits common extensor. More recently, with the advent of improved techniques and implants such as suture anchors, intraosseous screws and suspensory cortical buttons, single-incision techniques have once again gained popularity [14, 15].

At today’s date, there is still no consensus regarding which is the best surgical solution to approach distal biceps tendon rupture [16]. Some authors sustain that complication rate does not significantly differ between one and two-incision approaches (23,9% for one-incision procedures and 25,7% for two-incision procedures) [17]. Others claim that the double-incision has significantly lower complication rates than the single-incision-approach [18]. The objective of the present retrospective study was to describe the occurrence of the most frequent adverse events and clinical outcomes of patients undergoing surgical repair of distal biceps tendon with a modified double-incision technique. It was hypothesized that the double-incision approach represents a reliable surgical solution for distal biceps tendon rupture in well selected patients.

## Materials and methods

All distal biceps tendon ruptures undergoing surgical treatment in our department from January 2003 to January 2015 were considered eligible for study assessment. The inclusion criteria were as follows: age 18 years or above, acute or sub-acute tendon rupture (within 2 weeks from injury) treated with a modified double-incision surgical technique [19] and a minimum follow-up of 12 months. Only acute and sub-acute injuries were considered eligible because of proximal muscle retraction occurring in chronic ruptures [3]. We searched the department’s surgical electronic database using the following keywords: distal biceps tendon, distal biceps rupture. A total of 85 cases were found. Twenty-two patients were excluded, as they did not meet the inclusion criteria or refused to take part to study assessments.

All the operations were performed by two surgeons, both being highly experienced in elbow surgery. The cohort exclusively included male patients, with an average age of 44.8 years (min. 28 – max. 66 years). The dominant arm was involved in 39 cases (61.9%). At an average follow up of 24 months (min. 12 – max. 120 months) patients were clinically evaluated by measuring the degrees of pronation/supination, flexion/extension, documenting areas of hypoesthesia or neurological pain and asked to answer the Elbow Oxford Score (EOS), the Disabilities of Arm, Shoulder and Hand score (DASH) and the Mayo Elbow Performance score (MEPS). Patients’ overall satisfaction was recorded in a scale from 0 to 10.

Adverse events following surgical procedures were assessed and divided into two groups according to their frequency and severity, as described in the literature. Major complications included: persistent cramping or neurological pain, range of motion (ROM) deficiency > 30° in flexion-extension and pronation-supination compared to the healthy contralateral, isometric flexion strength deficiency > 30% and isometric supination strength deficiency > 60%, PIN palsy and non-traumatic re-rupture. Minor complications included: intermittent pain, ROM deficiency < 30° in flexion/extension and pronation/supination, isometric flexion strength deficiency < 30% and isometric supination strength deficiency < 60%, LACBN injury.

A digital Sauter FL dynamometer was used to test isometric muscle functioning in pronation/supination and flexion/extension with the elbow flexed at 90° and in full supination, with the aim to evaluate the strength of the injured joint. Results were compared with those achieved by the contralateral side, being compromised by the same injury in only one case. Patients with severe motion limitation were asked to undergo elbow radiographs.

### Surgical technique

**Fig. 1:**
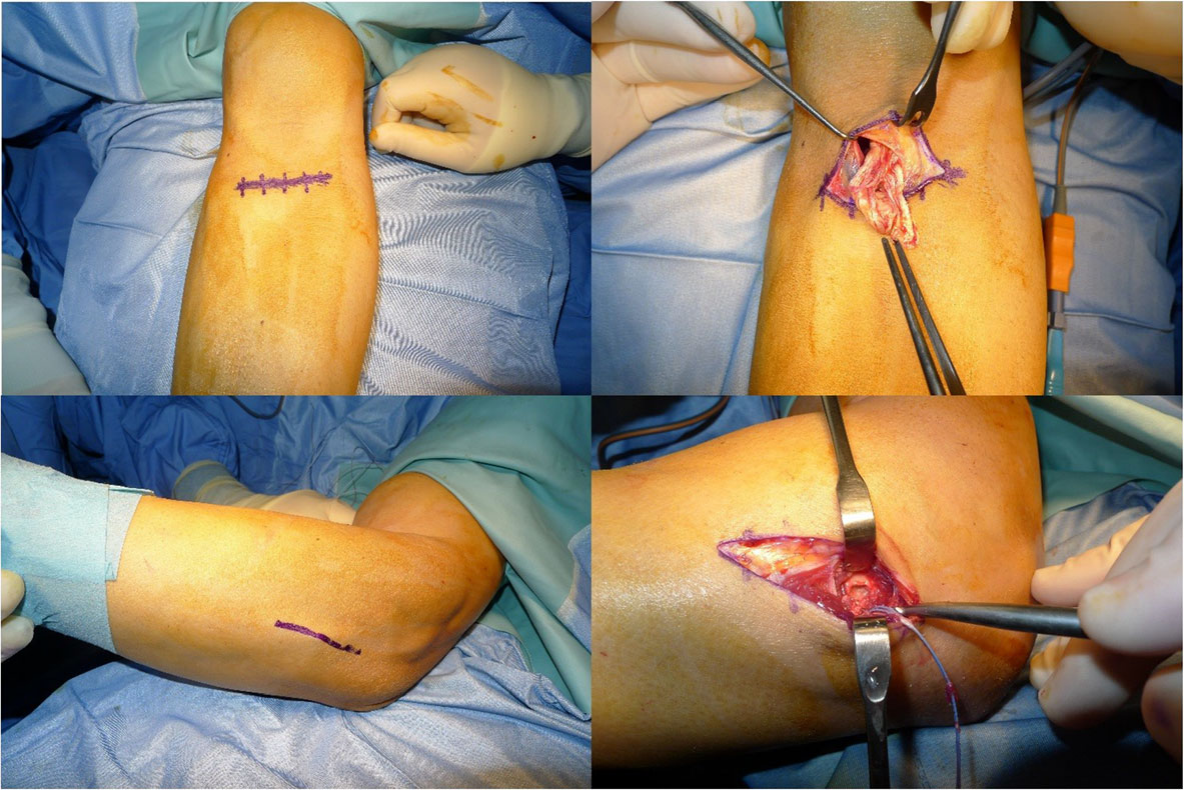
Some surgical steps: fist the anterior incision, followed by the finding of the distal tendon, then the crucial passage of the curved blunt hemostat in the biceps channel that point the place of the posterior incision. The radial tuberosity is then cleaned up from soft tissues and prepared with a high-speed burr, forming a 1.5 cm wide and 1 cm deep trench

With the patients lying in supine position, the tourniquet is applied to the injured arm. A minimally invasive, 3 cm transverse incision, over the antecubital fossa is made. After dissection of the subcutaneous tissue, particular care must be given to the lateral antebrachial cutaneous nerve (LABCN), discerning it from the biceps brachii muscle to avoid secondary traction. The muscle-tendon junction must be identified, and the stump tendon caught. The distal degenerated portion of the biceps tendon is resected, and two 3 cm-Krackow sutures are placed in the torn tendon. The radial tuberosity is palped with the index finger first and then using a blunt, curved hemostat that must be carefully inserted into the biceps channel. The instrument slips past the tuberosity and is advanced below, so its tip can be appreciated over the dorsal aspect of the proximal forearm placed in maximal pronation. The second incision is made over the tip of the instrument. The radial tuberosity is exposed by a lateral muscle-splitting technique by passing the instrument between extensor ulnaris carpi (EUC) and extensor digitorum communis (EDC), while the ulnar periosteum is never exposed. The radial tuberosity is then cleaned up from soft tissues and prepared with a high-speed burr, forming a 1.5 cm wide and 1 cm deep trench (Fig. 1). Three drill holes are placed approximately at 7–8 mm intervals through the dorsal cortical margin of the tuberosity. In this phase, accurate washing and sucking are mandatory to prevent heterotopic ossification caused by bone debris spreading. The tendon is passed through the second incision and carefully introduced into the trench prepared in the tuberosity.

**Fig. 2:**
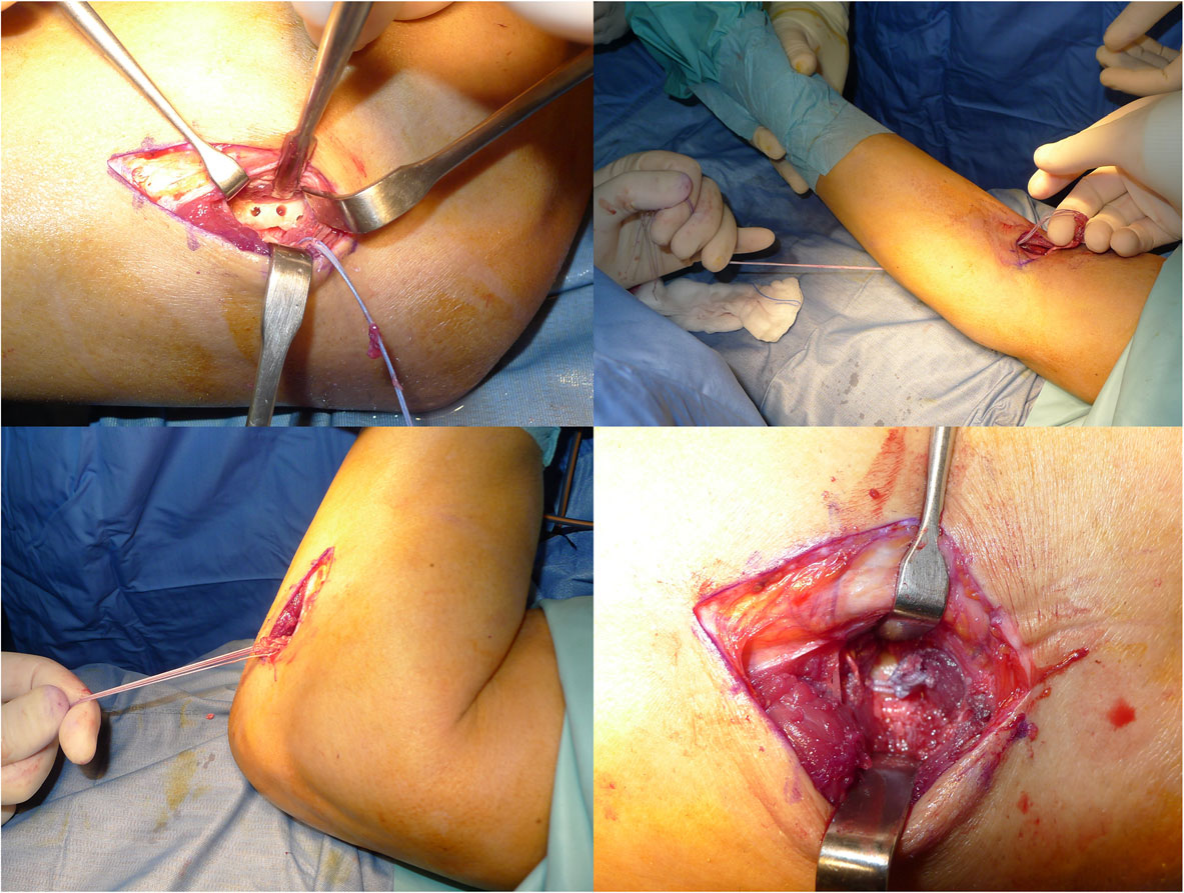
Final surgical steps: three drill holes are placed through the dorsal cortical margin of the tuberosity, the tendon is passed through the second incision and carefully introduced into the trench prepared in the tuberosity. Finally, with the forearm in the neutral position, the sutures are passed through the holes, pulled tight and tied

With the forearm in the neutral position, the sutures are passed through the holes, pulled tight and tied. A suction drain is placed in both wounds (Fig. 2). The elbow is then splinted at 90° of flexion, with the forearm at 45° of supination.

## Results

Sixty-three patients were considered eligible for assessment and were evaluated at an average of 24 months of follow-up (min. 12 – max. 120 months). Adverse events following the surgical procedure were divided into two groups: major and minor complications, according to their frequency and severity, as described in the methods section.

The recovery rate compared to the healthy contralateral was: 95% flexion (min: 110° - max: 135°; average 125°), 97% extension (min: − 2° - max: 15°, average: 2°), 88.5% supination (min: 0° - max: 90°; average 70°), and 92% pronation (min: 0° - max: 90°; average: 73°).

**Fig. 3:**
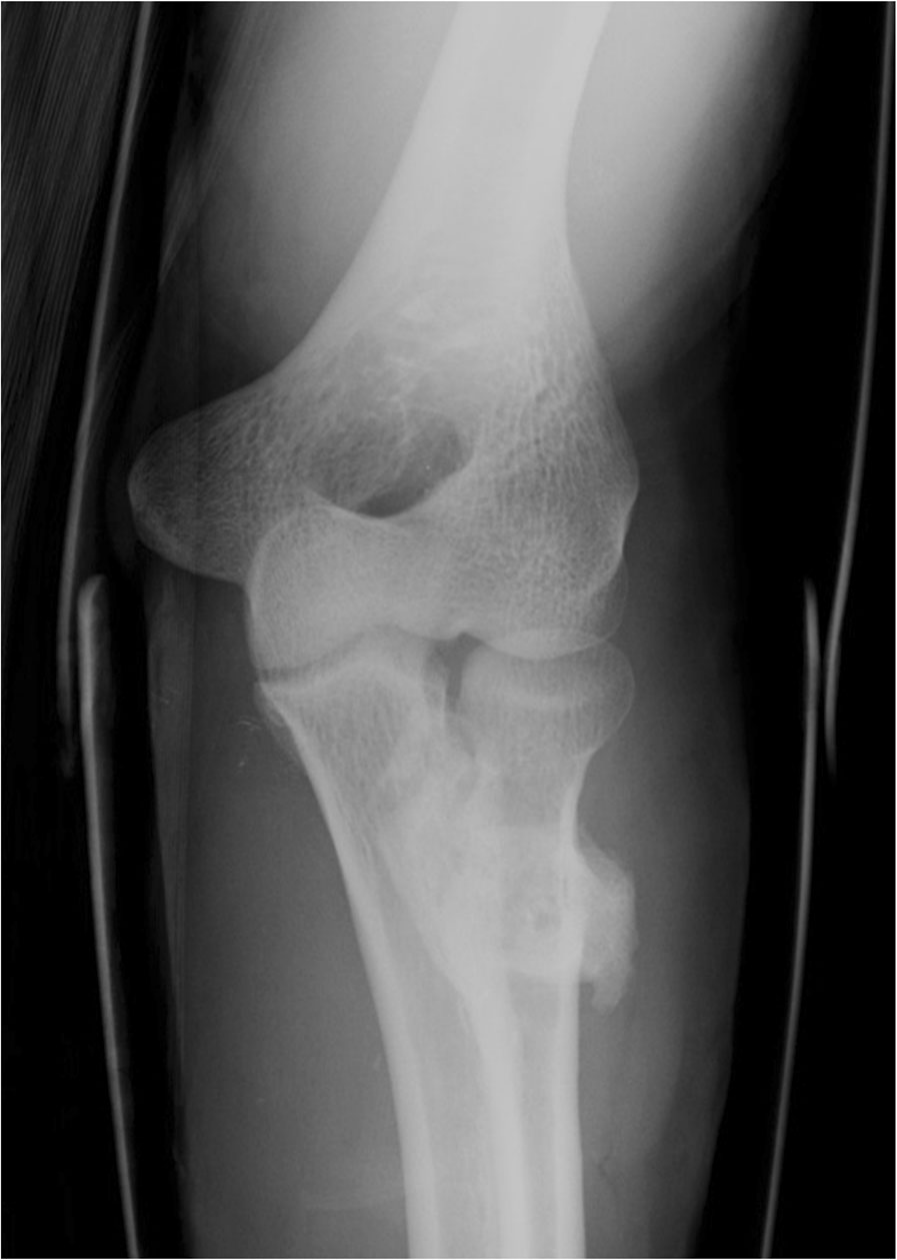
A case of proximal radio-ulnar synostosis with radiographic documentation

The reported major complications included: 1 (1.5%) case of proximal radio-ulnar synostosis with radiographic documentation (Fig. 3), 3 (4.5%) cases of PIN palsy and 1 (1.5%) case of non-traumatic tendon re-rupture. No cases of ROM deficiency > 30° were found.

The reported minor complications included: 6 (9.5%) cases of ROM defiency < 30°, 3 (4.7%) cases of LACBN injury, 3 (4.7%) cases of intermittent pain, 1 (1.6%) cases of flexion strength deficiency < 30% and 1 (1.6%) case of isometric supination strength deficiency < 60%, (Tab. 1).

**Table 1:** Patients’ case-series including dominant/non dominant forearm informations, follow-up visit, ROM and complication report. ROM values are expressed in degrees

N°	Age	Gender	Injured side	Follow up	ROM (ext-flex)	ROM (pron-sup)	Complications MAJOR	Complications MINOR
1	35	male	non dominant	12 months	0°-130°	90°-90°	no	no
2	42	male	dominant	15 months	0°-130°	90°-90°	no	intermittent pain
3	48	male	non dominant	2 years	0°-130°	90°-90°	no	no
4	62	male	non dominant	12 months	0°130°	90°-90°	no	no
5	43	male	non dominant	4 years	0°130°	90°-90°	no	no
6	28	male	dominant	19 months	0°-110°	80°-75°	no	ROM deficiency< 30°
7	37	male	non dominant	2 years	0°-130°	85°-90°	no	no
8	49	male	non dominant	5 years	0°-130°	90°-90°	no	no
9	66	male	dominant	1 years	0°-130°	75°-80°	no	ROM deficiency< 30°
10	30	male	dominant	8 years	0°-130°	90°90°	no	no
11	46	male	dominant	16 months	0°-130°	70°-50°	no	ROM deficiency< 30°
12	42	male	dominant	2 years	0°-130°	85°-90°	no	supination strength deficiency < 60%
13	62	male	non dominant	12 months	0°-130°	90°-90°	no	no
14	36	male	dominant	12 months	0°-130°	90°-90°	no	no
15	45	male	dominant	18 months	0°-130°	90°-85°	NIP transient palsy	no
16	59	male	dominant	15 months	0°-130°	90°-85°	no	no
17	48	male	dominant	18 months	0°-130°	65°-75°	no	ROM deficiency< 30°
18	39	male	dominant	2 years	0°-130°	85°-90°	no	no
19	37	male	non dominant	14 months	0°-130°	90°-90°	no	no
20	65	male	non dominant	4 years	15°-130°	90°-90°	no	no
21	52	male	dominant	2 years	0°-130°	90°-90°	no	no
22	59	male	non dominant	12 months	0°-130°	90°-90°	no	heterotopic ossifications
23	47	male	non dominant	16 months	0°-130°	90°-90°	no	no
24	42	male	non dominant	13 months	0°-130°	90°-90°	no	no
25	50	male	dominant	15 months	0°-130°	90°-90°	no	no
26	39	male	dominant	2 years	0°-130°	90°-90°	no	no
27	54	male	non dominant	12 months	0°-130°	90°-90°	no	no
28	47	male	non dominant	18 months	0°-130°	70°-80°	radio-ulnar synostosis	ROM deficiency< 30°
29	42	male	dominant	2 years	0°-130°	90°-90°	no	no
30	45	male	non dominant	19 months	0°-130°	90°-90°	no	intermittent pain
31	60	male	non dominant	16 months	0°-130°	90°-90°	no	no
32	36	male	non dominant	12 months	0°-130°	90°-85°	no	no
33	56	male	dominant	3 years	0°-130°	85°-90°	no	LACBN injury
34	47	male	dominant	12 months	0°-130°	90°-90°	no	no
35	40	male	dominant	14 months	0°-130°	90°-90°	no	no
36	54	male	non dominant	20 months	0°-130°	90°-90°	no	no
37	32	male	non dominant	4 years	0°-130°	90°-90°	no	no
38	42	male	dominant	2 years	0°-130°	90°-90°	no	no
39	36	male	dominant	13 months	0°-130°	90°-90°	no	no
40	40	male	dominant	12 months	0°-130°	75°-85°	atraumatic re-rupture	ROM deficiency< 30°
41	45	male	non dominant	2 years	0°-130°	90°-90°	no	no
42	57	male	dominant	17 months	0°-130°	90°-90°	no	no
43	39	male	dominant	16 months	5°-125°	85°-90°	no	heterotopic ossifications
44	36	male	dominant	10 years	0°-130°	90°-90°	no	no
45	50	male	dominant	12 months	0°-130°	90°-90°	no	no
46	54	male	dominant	15 months	0°-130°	90°-90°	no	no
47	41	male	dominant	2 years	0°-130°	90°-90°	no	no
48	36	male	dominant	12 months	0°-130°	90°-90°	no	intermittent pain
49	29	male	non dominant	20 months	0°-130°	90°-90°	no	no
50	46	male	dominant	19 months	0°-100°	85°-90°	NIP transient palsy	no
51	51	male	dominant	4 years	0°-130°	90°-90°	no	no
52	56	male	dominant	18 months	20°-125°	90°-85°	no	no
53	47	male	dominant	2 years	0°-130°	85°-90°	NIP transient palsy	LACBN injury
54	39	male	dominant	15 months	0°-130°	90°-90°	no	no
55	35	male	non dominant	18 months	0°-130°	90°-90°	no	flexion strenght deficiency < 30%
56	41	male	dominant	1 years	5°-130°	90°-90°	no	no
57	28	male	dominant	13 months	0°-130°	90°-90°	no	no
58	40	male	dominant	17 months	0°-130°	90°-90°	no	no
59	41	male	dominant	3 years	0°-130°	90°-90°	no	no
60	46	male	dominant	14 months	0°-125°	90°-90°	no	LACBN injury
61	51	male	non dominant	12 months	0°-130°	90°-90°	no	no
62	37	male	dominant	6 years	0°-130°	90°-80°	no	no
63	42	male	non dominant	16 months	0°-120°	90°-90°	no	no

NIP: posterior interosseous nerve, LACBN: lateral antebrachial coutaneous nerve, ROM: range of motion

The average DASH score was 8.5, OES resulted 41.5, MEPS overall score was 96.3 with a very good satisfaction (8.9/10) (Tab. 2).

**Table 2:** Clinical scores. Values are reported as mean, min. and max

Categories	Scores
M.E.P.S.	96.3 (min:70; max 100)
O.E.S	41.5 (min:17; max:48)
DASH score	8.5 (min: 1; max: 37,5)
Lickert scale	8.9 (min: 0; max: 10)

*MEPS* Mayo Elbow Performance score

*OES* Elbow Oxford Score

*DASH* Disabilities of Arm, Shoulder and Hand score

## Discussion

The rupture of the distal portion of the biceps tendon is not a very common tendon lesion. It occurs more often in a selected portion of middle-aged, male people, more frequently involving the dominant arm. Risk factors involved in this type of injury include smoke and use of drugs (antibiotics), but none of these has been identified as certain.

In the last decades, literature has shown the superiority of surgical treatment over non-operative management, demonstrating functional improvement in particular for supination strength recover [20]. Several surgical options have been described in literature: one incision-approach, using suture anchors, endobutton, biotenodesis screw for fixation, and a double-incision approach, using bone tunnels [8, 15, 19, 21, 22]. Standard and modified double incision approach differ one to each other in ulnar periosteum exposure, avoided by the Morrey’s muscle-splitting technique that reduces risk of synostosis [23, 24]. However, the minimal anterior incision on the cubital fossa, with muscle splitting technique, has not demonstrated to be a completely safe procedure to prevent the occurrence of nerve palsy (LACBN or radial) and heterotopic ossification. A recent comparison between the double-incision approach and the single-incision using endo-buttons, has demonstrated no significant differences between the two techniques in mean DASH score (6.31 versus 5.91, *p* = 0.697), mean Work DASH score (10.49 versus 0.93, *p* = 0.166), mean Sports DASH score (10.54 versus 9.56, *p* = 0.987) and complication rates (39.39% versus 32.0%, respectively) [25].

In their systematic review of 22 papers describing the treatment of acute distal biceps tendon repair, among which 4 studies describing both single and double-incision techniques, 14 studies involving the single incision and 4 studies the double-incision approach exclusively, Watson et al. reported a 23.9% complication rate for the single-incision technique and 25.7% complication rate for the double-incision approach. LABCN neuroapraxia was the most common complication overall (11.6% for one-incision and 5.8% for two-incision techniques); heterotopic ossification, stiffness and synostosis were more frequently reported in the two-incision technique (7.0%, 5.7% and 2.3% respectively) [17]. Grewal et al., evaluating mid-term outcomes of single and double-incision techniques reported significantly higher overall complication rate inthesingle-incision technique. Regarding heterotopic ossification, a single case was reported both in the single and double-incision groups [13].

Citak et al. compared the clinical and functional outcomes after distal biceps tendon repair using a single-incision approach with suture anchors and with a double-incision exposure using transosseous sutures. No statistically significant differences among groups were observed relative to ROM recovery rate. While no adverse events were described for the double-incision group, LACBN injury was reported in 5 cases in the single-incision cohort of patients [22].

Pairwise, Amin et al. conducted a meta-analysis of 87 articles, reporting higher frequencies of complications for the single-incision technique (performed with suture anchors, endobutton, biotenodesis screw), than for double-incision repair (performed with bone tunnels). Higher rates of nerve palsy (PIN, LACBN and radial nerve) and tendon re-rupture were reported in the single-incision group compared with the double-incision. On the other hand, heterotopic ossifications were described exclusively with the double-incision exposure.

As demonstrated by literature, advantages of the double-incision exposure include anatomic reinsertion on the radial tuberosity and consequent improved strength in supination and flexion [13], together with limited surgical costs. Limitations include higher rates of heterotopic ossifications.

In the present study including 63 subjects, the complications and clinical outcomes following the double-incision approach were examined and recorded in order to establish and determine appropriate indications for patients with acute ruptures of the distal biceps tendon. The obtained results were compared with those reported in literature relative to the surgical management of this injury.

Average ROM recovery showed excellent results compared to the healthy contralateral side, except from supination, which is the most impaired function in biceps tendon lesions [3, 22].

One case of radiographic radio-ulnar synostosis was observed in our series, determining complete block of prono-supination. The patient underwent surgical elbow arthrolysis with partial recovery of the limited movement. Three cases of transient PIN palsy, with complete recovery after 6 months, and 3 cases of transient LABC nerve palsy were reported in the examined cohort.

Relative to return at pre-injury activities, patients with high functional demand (sport professionals and manual workers) were found less satisfied than the majority of patients. Activities of daily living were possible for all the cohort, with an average DASH score of 8.5 and OES of 41.5. Complication rate and ROM recovery resulted comparable to available literature on surgical treatment of the same lesion (Tab. 3).

**Table 3:** Distal biceps tendon rupture surgical treatment as reported in literature, divided for single or double-incision approach. The rate of minor and major complications is reported

Study	Patients	Incision	Fixation method	ROM				Major complications	Minor complications
				Flexion	Extension	Pronation	Supination		
Tarallo et al. (present series)	63	2	Bone tunnels	125°	2°	73°	70°	3(4.5%) PIN transient palsy	3 (4.7%) Intermittent pain
				(min:110°-max: 135°)	(min:−2-max:15°)	(min:0°-max:90°)	(min:0°-max:90°)	1 (1.5%) Radio-ulnar synostosis	6 (9.5%) ROM deficiency < 30%
					97%	92%	88.50%	1(1.5%) Atraumatic re-rupture	1 (1.5%) Isometric flexion strength defiency< 30°
									1 (1.5%) Isometric supination strength defiency< 60°
									3(4.47%) LACBN injury
Grewal et al. ^(13)^	43	2	Suture anchors	131.8° ± 9.1	1.9° ± 4.6	72.4° ± 12.6	59.5° ± 11.5	1(2.32%) Atraumatic re-rupture	3(6.9%)LACBN injury
									1(2.32%) HO
	47	1	Bone tunnel	134.5° ± 6.9	3.0° ± 4.3	76.7° ± 8.2	63.9° ± 12.5	3(6.38%) Atraumatic re-rupture	19 (40.42%) LACBN injury
									1 (2.12%) HO
Gupta at al. ^(24)^	9	1	Endobutton	143°	0°	77°	81°	None	None
						(min:70°-max:82°)	(min:78°-max:85°)		
Citak et al. ^(25)^	15	1	Intraosseous screw	147°	1.3°	88°	89.3°	None	2(13.3%) LACBN injury
				(min:142.4°-max:150.7°)	(max:-0.6°-min:3.3°)	(min:85.7°-ma:90.3°)	(min:87.9°-max:90.8°)		
	24	1	Suture anchors	134°	1.3°	82.5°	81.7°	3(12.5%) re-rupture	3(12.5%) LACBN injury
				(min:122.6°-max:145.3°)	(min:-0.2-max:2.7°)	(min:76.2°-max:88.8°)	(min:74.9°-max:88.5°)		2(8.33%) ROM deficiency < 30%
	25	2	Bone tunnel	135°	1°	85.7°	84.7°	None	None
				(min:118.9°-max:151.1°)	(min:-0.6°-max:2.6°)	(min:78.6°-max:92.5°)	(min:77.9°-max:91.5°)		
Eardley et al. ^(26)^	14	1	Intraosseous screw	130°	0°	66°	74°	none	8(54%) LACBN injury
				(min:110°-145°)	(min:-10°-max:5°)	(min:50°-max:80°)	(min:50°-max:90°)		1(7.14%) HO
Johnson et al. ^(27)^	12	1	Suture anchors	142°	-2°	83°	85°	None	1(8.33%) LACBN injury
									1(8.33%) HO
	14	2	Bone tunnel	145°	0°	80°	83°	None	3(21.42%)HO
Oke A. Anakwenze et al. ^(28)^	12	2	Bone tunnel	153° ± 12.0°	0° ± 0°	78.5° ± 9.6°	78.9° ± 10.0°	None	none
Amin et al. ^(29)^	785	1	Suture anchors					17(2.1%) re-rupture	77(9.9%) LACBN injury
			Endobutton					13(1.7%)PIN palsy	25(3.2%) HO
			Biotenodesis screw						49(6.24%) intermittent pain
	498	2	Bone tunnel					3(0.6%) re-rupture	11(2.2%) LACBN injury
								11(2.2%) synostosis	36(7.2%) HO
								13(1.7%) PIN palsy	
David M. Weinstein et al. ^(30)^	32	1	Suture anchors	145° ± 8	0° ± 3	88° ± 10	73° ± 10	None	2(6.25%) LACBN injury
									1(3.12%) intermittent pain
Olsen JR et al. ^(31)^	20	1	Cortical button + interference screw	140° ± 6.2	7° ± 5.1	79° ± 6.8	72° ± 9.5	4(20%) PIN palsy	3(18%) LACBN injury
	17	1	Suture anchors	139° ± 5.6	5° ± 3.9	75° ± 9.2	76° ± 5.3	1(6%) PIN palsy	None

*PIN* posterior interosseous nerve

*ROM* range of motion

*HO* heterotopic ossification

*LACBN* lateral ante brachial cutaneous nerve

This study is not without limitations. The retrospective nature of the study design may have introduced selection bias and variations in treatment over time. In addition, being a single institution study may limit the generalizability of the results. Moreover, the mean follow-up was 24 months which, although adequate to determine results regarding pain relief, function and activity, may not be sufficient to draw conclusions regarding long term outcomes. No quantification of strength recovery in terms of Newton was reported and lastly, although post-operative MRI is described as a useful tool for tendon healing evaluation [23], no imaging examination was routinely performed in the study cohort.

On the other hand, strengths of the study include the large number of patients included, with the present series being the largest cohort in which clinical outcomes and complications of the double-incision technique in last decade’s literature have been described. Moreover, all patients were operated with a unique surgical technique, determining a large sample size to analyze its advantages and drawbacks.

## Conclusion

Although rate of complications and ROM recovery are similar among different surgical techniques, the Morrey-modified approach for distal biceps tendon repair represents a valid option to the single-incision techniques and finds its indication in young and active patients aiming to restore the pre-injury condition. Advantages of this approach include low costs and anatomical reinsertion, restoring flexion and supination strength. Surgery should be better performed within 2 weeks from injury to prevent proximal tendon retraction. To avoid frequent complications, including nerve palsy and severe ROM impairment, it’s recommended that only well-trained elbow surgeons approach this technique.
